# Chronic hepatitis B prevalence among Aboriginal and Torres Strait Islander Australians since universal vaccination: a systematic review and meta-analysis

**DOI:** 10.1186/1471-2334-13-403

**Published:** 2013-08-31

**Authors:** Simon Graham, Rebecca J Guy, Benjamin Cowie, Handan C Wand, Basil Donovan, Snehal P Akre, James S Ward

**Affiliations:** 1 The Kirby Institute, University of New South Wales, Sydney, Australia; 2 The Victorian Infectious Diseases Reference Laboratory (VIDRL), Melbourne, Australia; 3 Victorian Infectious Diseases Service, Royal Melbourne Hospital, Melbourne, Australia; 4 Department of Medicine, University of Melbourne, Melbourne, Australia; 5 Sydney Sexual Health Centre, Sydney Hospital, Sydney, Australia; 6 Baker IDI Heart and Diabetes Institute, Sydney, Alice Springs, Australia

**Keywords:** Indigenous, HBV, Sexually transmissible infection, STI, Hepatitis

## Abstract

**Background:**

In Australia, higher rates of chronic hepatitis B (HBsAg) have been reported among Aboriginal and Torres Strait Islander (Indigenous) compared with non-Indigenous people. In 2000, the Australian government implemented a universal infant/adolescent hepatitis B vaccination program. We undertook a systematic review and meta-analysis to assess the disparity of HBsAg prevalence between Indigenous and non-Indigenous people, particularly since 2000.

**Methods:**

We searched Medline, Embase and public health bulletins up to March 2011. We used meta-analysis methods to estimate HBsAg prevalence by Indigenous status and time period (before and since 2000).

**Results:**

There were 15 HBsAg prevalence estimates (from 12 studies) among Indigenous and non-Indigenous people; adults and pregnant women (n = 9), adolescents (n = 3), prisoners (n = 2), and infants (n = 1). Of these, only one subgroup (adults/pregnant women) involved studies before and since 2000 and formed the basis of the meta-analysis. Before 2000, the pooled HBsAg prevalence estimate was 6.47% (95% CI: 4.56-8.39); 16.72% (95%CI: 7.38-26.06) among Indigenous and 0.36% (95%CI:-0.14-0.86) in non-Indigenous adults/pregnant women. Since 2000, the pooled HBsAg prevalence was 2.25% (95% CI: 1.26-3.23); 3.96% (95%CI: 3.15-4.77) among Indigenous and 0.90% (95% CI: 0.53-1.28) in non-Indigenous adults/pregnant women.

**Conclusions:**

The disparity of HBsAg prevalence between Indigenous and non-Indigenous people has decreased over time; particularly since the HBV vaccination program in 2000. However HBsAg prevalence remains four times higher among Indigenous compared with non-Indigenous people. The findings highlight the need for opportunistic HBV screening of Indigenous people to identify people who would benefit from vaccination or treatment.

## Background

The hepatitis B virus (HBV) is a blood borne virus which can lead to liver failure and cancer of the liver [1]. HBV can be transmitted through contact with infected blood and body fluids (unsafe injecting and sexual transmission) [2], and through vertical transmission from mother to child during birth [3]. HBV testing can provide results for HBV surface antibodies, HBV core antibodies and HBV surface antigen (HBsAg) [1]. Serology can determine if a person has chronic infection, is susceptible to infection or is immune through vaccination or past infection. Chronic HBV infection is defined as the presence of HBsAg for greater than six months [4]. HBsAg was first discovered in 1965, in an Aboriginal Australian male and was originally known as the Australia antigen [5].

The World Health Organization classifies a HBsAg population prevalence of 8% or greater as high, 2-8% as intermediate and 2% or less as low [6]. In Australia, an estimated 218,000 (1.0% population prevalence) people were living with chronic HBV in 2011 [7]. Aboriginal and Torres Strait Islander (hereafter referred to as ‘Indigenous’) people represent 2.6% of the Australian population, however they account for an estimated 10% of those living with chronic HBV [7,8]. Among Indigenous Australians, a variety of modes of HBV transmission are believed to have contributed to high levels of chronic HBV. However it is likely that a higher proportion of infections have historically occurred at birth or early in life [9], resulting in a higher prevalence of chronic HBV infection due to the increased risk of progression to chronicity during childhood infections [10]. Australia, has high levels of antenatal HBV screening and since the implementation of universal infant vaccination including a birth dose since 2000, transmission of HBV from mother to child has decreased [11,12].

In Australia, it is estimated that nearly half of those living with chronic HBV remain undiagnosed [7], and less than 3% are currently receiving antiviral treatment [13]. An estimated 15-40% of people living with untreated chronic HBV develop complications, including cirrhosis and/or hepatocellular carcinoma (HCC), which is now the fastest increasing cause of cancer death nationally [14,15]. The incidence of HCC is between two and eight times greater among Indigenous compared with non-Indigenous people [16]. A greater burden of other diseases such as diabetes, sexually transmitted infections and renal disease have been reported among Indigenous compared with non-Indigenous people and may contribute to the Indigenous population’s vulnerability to HBV infection [17,18].

The HBV vaccine has been available in Australia since 1982 [6]. The HBV vaccine is one of the most effective ways to prevent infection and can reduce perinatal transmission by up to 90% [19]. It is also estimated that between 85-90% of HBV related deaths are vaccine-preventable [20]. In 1985, the Northern Territory (NT), (a state with approximately 30% of its population identifying as Indigenous) introduced HBV screening to all pregnant women and vaccination to infants born to mothers living with chronic infection. In 1990, universal infant vaccination was implemented, and in 1998 a catch-up program targeting 6-16 year olds was introduced [10]. In 2000, HBV vaccination of all infants commenced in other states and territories of Australia and the introduction of a universal adolescent (teenagers aged 12-15 years) school based HBV vaccination catch-up program commenced in 1998 [20].

In 2010, Australia’s first National Hepatitis B Strategy 2010-2013 [8] was released and recommended HBV vaccination among priority population groups including people from culturally and linguistically diverse populations, children born to mothers with chronic HBV infection and Indigenous people. National guidelines released by the National Aboriginal Community Controlled Health Organisation (NACCHO) and the Royal Australian College of General Practitioners recommend HBV screening of Indigenous people who have not been vaccinated or vaccination status is unknown as well as to people at ‘high risk’ of blood borne viruses (BBV) [10,21].

In 1996, a HBV review provided prevalence estimates of HBsAg in Australia and aimed to provide evidence for the development of a HBV vaccination policy. This review estimated a HBsAg prevalence among Indigenous pregnant women of 10% [22]. Since 1996, a number of studies have reported HBsAg prevalence among Indigenous people and in 2000 Australia implemented a universal infant and adolescent HBV vaccination program. We undertook a systematic review to assess if the disparity in HBsAg prevalence between Indigenous and non-Indigenous people has decreased over time, especially since 2000. This review also provides an up-to-date picture of the burden of HBsAg among Indigenous people and will help to inform current screening policies.

## Methods

### Setting

In 2011, the Australian Bureau of Statistics (ABS) estimated that there were 548,370 Indigenous people (2.6% of the Australian population) [23]. The median age of the Indigenous population was 21 compared with 37 years in non-Indigenous people [24]. The number of Indigenous people varied across the states and territories of Australia with the largest population residing in New South Wales (152,700) followed by Queensland (144,900) [24]. The ABS uses the Australian Standard Geographical Classification (ASGC) to define whether a geographical area in Australia is urban, regional or remote. This allows the Australian government to develop different policies suitable to people living in regional or remote areas as they often have less access to services such as specialist health care [25]. A higher proportion of Indigenous compared with non-Indigenous people live in regional and remote areas [18]. In addition to general practice clinics in Australia, there are an estimated 142 primary health care centres known as Aboriginal Community Controlled Health Services (ACCHS) which provide culturally appropriate medical and allied health care to Aboriginal people.

### Literature search

This review was conducted according to the PRISMA statement [26]. The electronic databases Medline and Embase and public health bulletins were searched in March 2011. Reference lists of studies were also examined for relevant papers.

The following MeSH search terms (and variations) were used:

1. Hepatitis B or hepatitis B surface antigen or HBV or HBsAg; and

2. Aboriginal or Indigenous; and

3. Australia.

The studies were reviewed and information was extracted by the lead author. A study was included if it presented HBsAg prevalence among Indigenous Australians. Studies were excluded if they reported HBsAg prevalence in non-Indigenous people only, were reviews or commentaries, or did not present primary data. For each study that met the inclusion criteria, information was extracted on; study period, state/territory, study design, sex, age group, Indigenous status, sample size, HBsAg prevalence and 95% confidence intervals (CIs) (Table 1). The studies were separated into two time periods, before 2000 and since 2000 when universal infant and adolescent school based HBV vaccination programs commenced in the majority of states and territories of Australia. The following sub-groups were identified across the different studies, adults, adolescents/teenagers, pregnant women, prisoners, and infants. The authors defined adolescents/teenagers as those aged between 9-19 years and infants as those aged 0-1 year of age. If studies did not present 95% confident intervals (CI), the authors calculated them using the Exact Binomial Method in STATA 12 statistical software (STATA Corporation, College Station TX) [27].

**Table 1:** HBsAg prevalence estimates among indigenous and non-indigenous Australians by study population and before and since 2000

**Pre/ post 2000**	**Study population**	**Author &year published**	**Study period**	State/ territory*	**Study design**	**Sex**^⌶^	**Age group (yrs)** +	**Indigenous status**	**Sample size (n)**	**HBsAg prevalence (%)**	**95% CI**
Pre	Adolescents	Barrett [29], 1972	1972	QLD & NT	Cross sectional survey of blood samples	M,F	N/S	Indigenous	731	3.41	2.23-5.01**
Pre	Adults	Barrett [29], 1972	1972	QLD & NT	Cross sectional survey of blood samples	M,F	N/S	Indigenous	160	8.13	4.40-13.49**
Pre	Adults	Barrett [30], 1976	1976	QLD & NT	Cross sectional survey of blood samples	M,F	N/S	Indigenous	662	6.90	5.13-9.16**
								Non-Indigenous	234	0.38	0.01-2.36**
Pre	Adults	Burrell [31], 1983	1979-1982	SA	Cross sectional survey of blood samples	M,F	N/S	Indigenous	327	25.99	21.32-31.10*
								Non-Indigenous	22,800	0.20	0.15-0.30**
Pre	Adolescents	Campbell [32], 1985	November 1985	NSW	Cross sectional Community survey	M,F	12-16	Indigenous	89	12.02	6.33-21.04*
Pre	Pregnant women	Moore [33], 1987	1983-1985	WA	Cross sectional Midwifery analyses	F	N/S	Indigenous	817	3.60	2.39-7.88**
Pre	Adults	Holman [34], 1987	May-Sept 1986	WA	Cross sectional Community survey	M,F	N/S	Indigenous	1,150	7.90	6.42-9.63**
Pre	Adolescents	Holman [34], 1987	May-Sept 1986	WA	Cross sectional Community survey	M,F	12-19	Indigenous	177	4.52	1.97-8.71**
Pre	Adults	Campbell [36], 1989	Nov 1985	NSW	Cross sectional Community survey	M,F	N/S	Indigenous	375	19.20	15.34-23.56**
								Non-Indigenous	268	2.01	0.61-4.30
Pre	Adolescents	Gill [35], 1990	1989	WA	Cross sectional School survey	M,F	10-19	Indigenous	186	6.1	3.8-9.5**
								Non-Indigenous	301	0.3	0.1-1.8**
Pre	Adolescents	Campbell [36], 1989	1992	NSW	Cross sectional Community survey	M,F	12-16	Indigenous	297	14.01	10.39-18.63
								Non-Indigenous	111	0.0	0.0-0.0
Pre	Adults	Patterson [37], 1993	1983-1984	NSW	Cross sectional Community survey	M,F	N/S	Indigenous	236	16.91	12.39-22.36**
								Non-Indigenous	268	2.01	0.61-4.30**
Pre	Adults	Patterson [37], 1993	1987-1988	NSW	Cross sectional Community survey	M,F	N/S	Indigenous	212	5.19	2.62-9.09**
								Non-Indigenous	422	0	0.0-0.0**
Pre	Infants	Campbell [32], 1989	1992	NSW	Cross sectional Community survey	M,F	0-1	Indigenous	49	22.45	3.89-31.66**
								Non-Indigenous	11	9.09	
Pre	Adolescents	Gardner [38], 1992	1992	NT	Cross sectional school survey	M,F	9-13	Indigenous	439	8.20	5.81-11.17**
								Non-Indigenous	665	0.41	0.09-1.31**
Pre	Adolescents	Malcolm [39], 2000	1999	QLD	Cross sectional Community survey	M,F	N/S	Indigenous	108	26.02	17.97-35.25**
Post	Pregnant women	Panaretto [40], 2006	2000-2003	QLD	Longitudinal cohort	F	15-40	Indigenous	419	0.81	0.15-2.08
Post	Pregnant women	Wood [41], 2008	2002-2004	NT	Cross sectional Midwifery analyses	F	10-50	Indigenous	522	5.50	3.75-7.88
								Non-Indigenous	516	0.79	0.21-1.97
Post	Pregnant women	Romanes [42], 2006	2003	NT	Cross sectional clinical audit	F	15-47	Indigenous	540	4.09	2.57-6.10
							15-47	Non-Indigenous	862	1.19	0.56-2.12
Post	Pregnant women	Schultz [43], 2007	2005	NT	Cross sectional clinical audit	F	N/S	Indigenous	432	3.21	1.78-5.37
							N/S	Non-Indigenous	359	0.61	0.07-1.98
Post	Pregnant women	Schultz [44], 2008	2003 & 2005	NT	Cross sectional data analysis	F	15+	Indigenous	973	3.70	2.60-5.09**
							N/S	Non-Indigenous	1221	0.90	0.45-1.61
Post	Prisoners	van der Poorten [45], 2008	2002-2005	NSW	Cross sectional prison survey	M	12-19	Indigenous	179	3.41	1.30-7.15**
								Non-Indigenous	530	1.09	0.42-2.45**
Post	Prisoners	Templeton [46], 2010	2000-2004	NSW	Cross sectional audit	M	14-20	Indigenous	79	11.39	6.29-19.85
Post	Prisoners	Gilles [47], 2008	2006	WA	Cross sectional clinical audit	M,F	18-50	Indigenous	155	3.24	1.06-7.37
							18-50	Non-Indigenous	30	3.33	0.08-17.22
Post	Adults	Carroll [48], 2010	2008	QLD	Cross sectional clinical audit	M,F	15-54	Indigenous	112	0.93	0.02-4.87
Post	Adults	Carroll [49], 2010	2008	NT	Cross sectional clinical audit	M,F	15-54	Indigenous	112	11.96	6.33-19.03
Post	Adolescents	Dent [50], 2005	May-July 2005	NT	Cross sectional Community survey	M,F	14-15	Indigenous	37	11.08	3.03-25.42

*Northern Territory (NT), New South Wales (NSW), Queensland (QLD), South Australia (SA). ^⌶^Males (M), Females (F). + N/S not stated. **Authors calculated 95% confidence intervals (Binomal Exact Method).

### Meta-analysis

**Figure 1:**
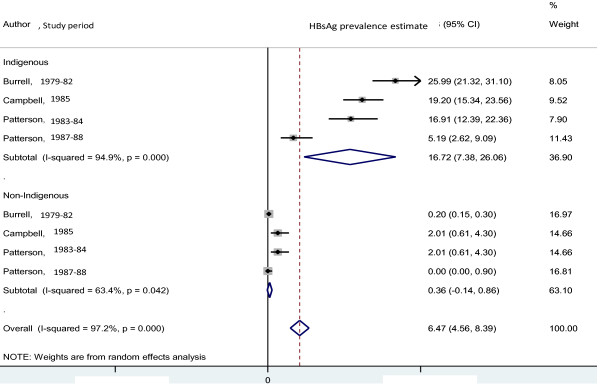
HBsAg prevalence before 2000, by author, study period among Indigenous compared with non-Indigenous adults.

**Figure 2:**
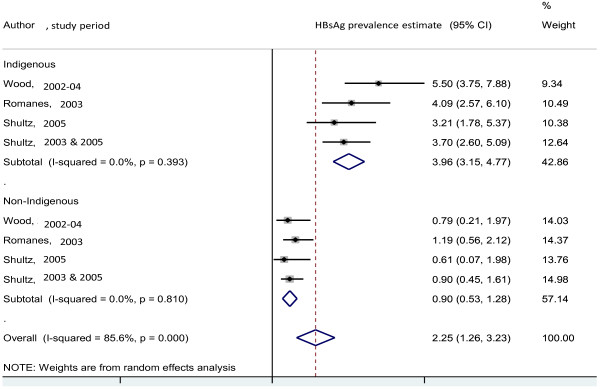
HBsAg prevalence since 2000, by author, study period among Indigenous compared with non-Indigenous pregnant women.

A meta-analysis was conducted to estimate the HBsAg prevalence by Indigenous status and time period (before 2000 compared with since 2000). The meta-analysis focused on sub-groups with studies conducted before and since 2000, and each study provided HBsAg prevalence estimates for both Indigenous and non-Indigenous people. Only adults and pregnant women combined met this criteria. The meta-analysis was conducted using weighted inverse variance methods, the DerSimonian-Laird method [28] assuming a random-effects model. We used the *I*^2^ test to estimate the approximate proportion of total variability in point estimates that could be attributed to heterogeneity other than that due to chance. The results were presented as forest plots (Figures 1 and 2).

## Results

### Studies

**Figure 3:**
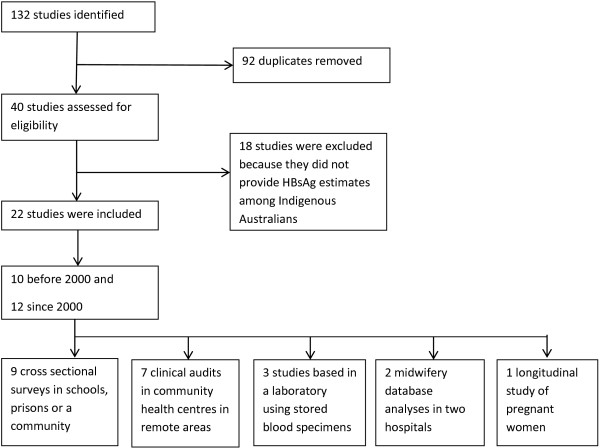
Flow diagram of studies included in the review.

There were 22 [29-50] studies included in the review (Figure 3), representing five states/territories of Australia (Table 1). Of the 22 studies: nine (41%) studies were conducted in remote areas, six (27%) in regional areas, and seven (32%) did not specify a region in which the study was conducted or were data analyses using patient or midwifery databases. Ten studies were conducted before 2000 and 12 since 2000 (Table 1).

The most common study design of the 22 studies were cross-sectional surveys conducted in schools, prisons or a community (n = 9). Other studies were, clinical audits conducted in community health centres (n = 7), serology testing of a convenient sample of stored blood specimens (n = 3), retrospective analyses of midwifery databases (n = 2) and a longitudinal study examining sexual health screening of pregnant women attending an ACCHS (n = 1).

The 22 studies provided 28 HBsAg prevalence estimates among Indigenous people. The studies presented HBsAg prevalence in a number of sub-groups. A study by Campbell and colleagues [32] presented HBsAg prevalence by age groups and as a result we were able to present HBsAg prevalence among Indigenous infants, adolescents and adults. A study by Patterson and colleagues [37] presented HBsAg prevalence among Indigenous adults in two time periods, 1983-1984 and 1987-1988 and Holman and colleagues [34] presented HBsAg prevalence among Indigenous adolescents and adults. The 28 HBsAg estimates among Indigenous people were in the following sub-groups; adults (n = 9), adolescents/teenagers (n = 8), pregnant women (n = 6), prisoners (n = 3), and infants (n = 2).

Of the 22 studies, 12 studies provided 15 HBsAg prevalence estimates among both Indigenous and non-Indigenous people in the following sub-groups; adults (n = 5), pregnant women (n = 4), adolescents (n = 3), prisoners (n = 2) and infants (n = 1).

### HBsAg prevalence in individual studies before 2000

Before 2000, six of the ten studies reported a HBsAg prevalence among Indigenous people of 8% or greater (considered high by WHO). The highest HBsAg prevalence (26%) was reported by two studies; one was a cross sectional survey by Burrell and colleagues in 1979-82 of stored blood samples [31] and the other an investigation of a cluster of HBsAg seropositive cases in northern Queensland among Indigenous adolescents [39]. There were six studies in adults and pregnant women; three were community-based surveys, and three serology testing of a convenient sample of stored blood specimens. Six of the ten studies presented HBsAg prevalence for both Indigenous and non-Indigenous people and the prevalence estimates were higher among Indigenous compared with non-Indigenous adults, pregnant women and adolescents. The greatest disparity was in the study by Burrell and colleagues [31] which found HBsAg prevalence among Indigenous adults of 26% compared with 0.2% in non-Indigenous adults. Two studies [32,38] (conducted in 1985 and 1989) presented breakdowns by sex, and found the HBsAg prevalence among Indigenous adults and infants, was higher in males compared with females. A study by Gardner and colleagues [38] in the NT (conducted in 1989), found an overall HBsAg prevalence among Indigenous adolescents aged 9-17 years of 8.2%, however the prevalence was higher among Indigenous children living in remote compared with urban areas. Nine of the ten studies conducted before 2000, were actually conducted before 1990.

### HBsAg prevalence in individual studies since 2000

Since 2000, nine of twelve studies reported HBsAg prevalence among Indigenous people between 2-8% (considered intermediate by WHO); the highest estimate was 12% from a study in 2008 which was a clinical audit of adults conducted in a remote ACCHS in the NT. Three other studies in the NT aimed to provide more population-based estimates. The first by Schultz and colleagues [44] estimated antenatal population-based HBsAg prevalence in the NT by reviewing over 2000 records from two hospitals (in Darwin and Alice Springs). These two hospitals were responsible for delivering 71% of Indigenous births in 2003 and 2005 and found the HBsAg prevalence among Indigenous pregnant women was 4%. The second study by Wood and colleagues [41] involved data linkage of the NT Midwives database and HBV serology data and found the HBsAg prevalence was 5.5% among Indigenous pregnant women. The study by Wood and colleagues reported the HBsAg prevalence among pregnant women by age groups and found the prevalence was lowest in 15-19 year olds, and highest in 35-39 year olds. The third study by Romanes and colleagues [42] in 2003 involved a clinical audit of medical records at the Royal Darwin Hospital and found the HBsAg prevalence among Indigenous pregnant women was 4.1%. There was one further study in pregnant women [40], conducted at an ACCHS in Northern Queensland between 2000-2003. The study was an intervention program aimed at improving sexual health screening during pregnancy, and found a low HBsAg prevalence of 0.8%. Three of the studies in pregnant women reported very high HBsAg screening rates (greater than 90%).

Three further studies involved clinical audits, with two of the three [45,47] comparing HBsAg prevalence among Indigenous and non-Indigenous prisoners. These two studies in prisoners had small samples sizes (101 and 185), and were conducted mostly in males (>80%), with one study showing a higher HBsAg prevalence among Indigenous (3%) compared with non-Indigenous prisoners (1%) and the other found a similar prevalence of 3% among Indigenous and non-Indigenous prisoners. The third study focused on adolescents (n = 37) attending a remote health service and found a HBsAg prevalence of 11%. There were no HBsAg prevalence studies since 2000 among infants.

Since 2000, there were six studies which presented a HBsAg prevalence for both Indigenous and non-Indigenous people with five estimating a higher HBsAg prevalence among Indigenous compared with non-Indigenous people.

### Meta-analysis

Before 2000, the pooled HBsAg prevalence estimate was 6.47% (95%CI:4.56-8.39; I^2^ = 97.2%, p < 0.001, 3 studies); 16.72% (95%CI:7.38-26.06; I^2^ = 94.9%, p < 0.001) among Indigenous compared with 0.36% (95%CI:-0.14-0.86; I^2^ = 63.4%, p = 0.042) in non-Indigenous adults/pregnant women (Figure 1).

Since 2000, the pooled HBsAg prevalence estimate was 2.25%, (95%CI: 1.26-3.23; I^2^ = 85.6%, p < 0.001) (4 studies); 3.96% (95%CI:3.15-4.77 I^2^ = 0%, p = 0.393) among Indigenous compared with 0.90% (95%CI:0.53-1.28; I^2^ = 0%, p = 0.810) in non-Indigenous adults/pregnant women (Figure 2).

## Discussion

The disparity in HBsAg prevalence between Indigenous and non-Indigenous Australians appears to have decreased over time, especially in studies conducted since 2000 compared with before 2000. This coincides with the introduction of universal vaccination programs in Australia, for adolescents (since 1998) and infants (since 2000). However in studies conducted since 2000, the prevalence of chronic HBV remains four times higher among Indigenous compared with non-Indigenous people.

This review found a higher HBsAg prevalence among Indigenous pregnant women compared with non-Indigenous pregnant women in studies conducted since 2000 (mainly in the NT) and the study by Wood and colleagues [41] found HBsAg prevalence was slightly lower in 15-19 year olds pregnant women, an age group more likely to have been vaccinated. These findings highlight the need for ongoing antenatal screening of pregnant women living in remote Indigenous communities and vaccination to not only decrease chronic HBV prevalence among pregnant women but also perinatal transmission from mother to child [19].

The higher HBsAg prevalence found among Indigenous compared with non-Indigenous adults highlights the importance of offering opportunistic HBV screening to Indigenous people. In 1990, New Zealand (NZ) introduced a HBV screening program in response to the higher HBV prevalence in Maori, Pacific Islander and Asian populations compared with other New Zealanders [51]. The program was based primarily on supporting general practitioners (GPs) and Maori and Pacific Islander providers to recruit individuals for opportunistic HBV testing, by invitation letter or phone call, or through community promotion, community meetings or churches. In a three-year period between 1999 and 2002 a total of 177,328 people were tested for HBV; 153,605 (87%) were Maori, Pacific Islander or Asian adults, and the program reached 28%, 35% and 20% of the Maori, Pacific Islander and Asian adult target population, respectively. In Australia, general practitioners and ACCHS are ideally placed to undertake opportunistic HBV screening, as studies have shown that the majority of Indigenous people attended ACCHS for their health care and health education needs [52].

HBV screening also identifies those in need of vaccination, treatment and management. Appropriate treatment of chronic HBV has been found to substantially reduce the risk of liver cancer [53] and is cost effective in the Australian context [54]. Systematic liver cancer surveillance in NZ has shown to increase the survival of people enrolled in the HBV screening programme [55,56].

There are some limitations to be considered when interpreting our findings. Many of the studies were clinical audits conducted in community health centres or ACCHS and thus the findings could be influenced by the screening protocols in these health services. This could potentially over-estimate HBsAg prevalence if clinicians are preferentially testing those with symptoms or at higher risk of infection rather than conducting universal screening. That being said, in a number of studies among pregnant women the testing uptake was consistently over 90%. Furthermore, many of the studies were conducted in regional or remote areas, so results may not be generalisable to Indigenous people living in urban areas of Australia. With respect to included studies, we did not search all the grey literature so it is possible that some studies were not identified. In addition, although the comparison of HBsAg prevalence before and since 2000 via meta-analysis was undertaken, the findings may be influenced by different study designs and populations included in these periods. Finally, we were unable to conduct a meta-analysis for a number of sub-groups due to the limited number of studies available.

## Conclusion

Although HBsAg prevalence appears to have decreased over time in Australia, it remains four times higher among Indigenous compared with non-Indigenous people. Our findings suggest the need for opportunistic HBV screening of Indigenous people to identify those who need vaccination and those needing treatment and regular monitoring.

## Competing interests

The authors declare that they have no competing interests.

## Authors’ contributions

SG wrote the manuscript, collected, analysed and interpreted the data from each study. RG provided guidance on data analysis and contributed to writing. BC provided guidance into the clinical interpretation of the studies and the recommendations. HW conducted the meta-analysis. BD contributed to the writing. SA contributed to the writing. JW conceived the paper and contributed to the writing. All authors read and approved the final manuscript.

## Pre-publication history

The pre-publication history for this paper can be accessed here:

http://www.biomedcentral.com/1471-2334/13/403/prepub
